# Supplementary material for: An updated assessment of Symbiodinium spp. that associate with common scleractinian corals from Moorea (French Polynesia) reveals high diversity among background symbionts and a novel finding of clade B
Source: PeerJ. 2017 Jan 5;5:e2856. doi: 10.7717/peerj.2856 (PMC5289445; doi:10.7717/peerj.2856)
Supplement: Supplemental Information 1 [file peerj-05-2856-s001.docx]

**SUPPLEMENTARY METHODS**

The estimation of DNA copy number was evaluated using purified PCR products targeting the 28S ribosomal DNA region from each cultured *Symbiodinium* clade A-F. PCR products were obtained with universal primers for clades A-D and F (~580 bp) (Richter *et al.* 2008), and for clade E (830 bp) (Wilcox 1998). The PCR products were then mixed together at the same concentration using a NanoDrop® ND-1000 spectrophotometer. The mix contained the six universal clade 28S PCR products (Table S2), which were initially concentrated at 1 pg per qPCR reaction for each clade (equivalent to 1.73.10^6^ copy 28S for clade A-D and F, and 1.21•10^6^ copy 28S for clade E). 28S copy number estimation was calculated using DNA concentration (C in g), the molecular weight (MW) of a double-standed DNA molecule (660 bp), the size (S) of the PCR amplicon (580 bp), and the Avogadro constant corresponding to 1 mole (Mole: 6•10^23^) according to the formula: 28S copy number = [C/(MW x S)] x Mole.

The counting of ‘wild’ *Symbiodinium* cells (freshly isolated from corals by centrifugation of total coral tissue and resuspended in 0.45 µm-filtered sweater) previously identified with symbiotic mono-clade communities, was performed in order to validate the utility of the numeration method based on 28S copy number. The counting of *Symbiodinium* cells from clades A, C and D (clade B never detected in mono-clade communities) was performed in 0.2 mL-PCR tubes using a Malassez counting chamber in order to generate a series of known cell density aliquots (A: 36-3612 cells, C: 276-4135 cells and D: 225-4507 cells). The tubes were centrifuged to discard the seawater, and the pellet resuspended with 50 µL of sterilized water. For each of the cell aliquots, three cycles were applied: (1) 7 min at 98°C with intermittent vortexing (20 sec every 3 min), (2) 5 min in dry ice, and (3), directly after these steps, 50 µL of sterilized water was added to the aliquots. Each *Symbiodinium* cell extract was then used for qPCR quantification. The mono-specific composition of each *Symbiodinium* cell mixture from clades A, C and D was confirmed by a positive amplification of only the corresponding clade with its clade-specific primer, and no amplification with the other primer sets. Standard curves were constructed using a series of five known cell densities and plotted as Ct values versus the logarithm of the corresponding cell number (Fig. S2).
