# Supplementary figures and images for: An updated assessment of Symbiodinium spp. that associate with common scleractinian corals from Moorea (French Polynesia) reveals high diversity among background symbionts and a novel finding of clade B
Source: PeerJ. 2017 Jan 5;5:e2856. doi: 10.7717/peerj.2856 (PMC5289445; doi:10.7717/peerj.2856)

a.

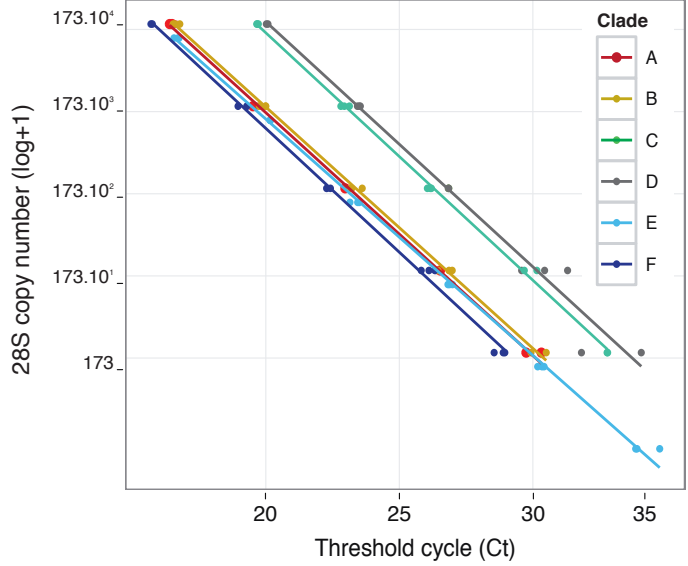

b.

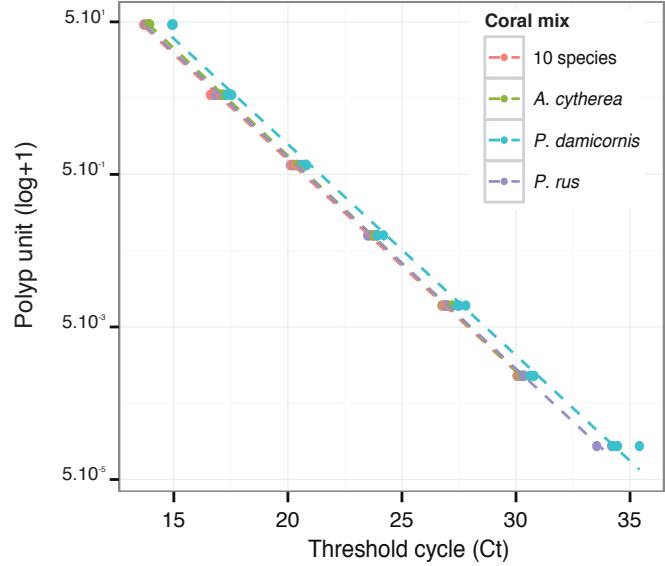

Supplement: Figure S1 — Standard curves for different primers, corresponding to Ct values versus logarithmic 10 fold dilution of purified 28S PCR products. Each plot corresponds to individual Ct values obtained from three technical replicates. Primer efficiencies were deduced by linear regression: (A) mix of purified 28S PCR products from Symbiodinium A–F each concentrated at equal concentration, 1⋅1011×(−0.681x) (clade A, R2 = 0.999), 1⋅1011 × e(−0.676x) (clade B, R2 = 0.999), 1⋅1012 × e(−0.687x) (clade C, R2 = 0.983), 1⋅1012 × e(−0.694x) (clade D, R2 = 0.998), 6⋅1010 × e(−0.663x) (clade E, R2 = 0.998) and 8⋅1010 × e(−0.694x) (clade F, R2 = 0.997); and (B) mix of coral DNA each concentrated at equal concentration, universal coral primer set, 1.25⋅104 × e(−0.694x) (mix of 10 coral species: R2 = 0.999), 9.02⋅105 × e(−0.705x) (mix of A. cytherea: R2 = 1.000), 6.58⋅105 × e(−0.692x) (mix of P. rus: R2 = 0.999) and 1.0⋅106 × e(−0.629x) (mix of P. damicronis: R2 = 0.996). [file peerj-05-2856-s003.pdf]

Clade

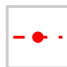

A

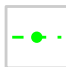

C

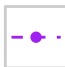

D

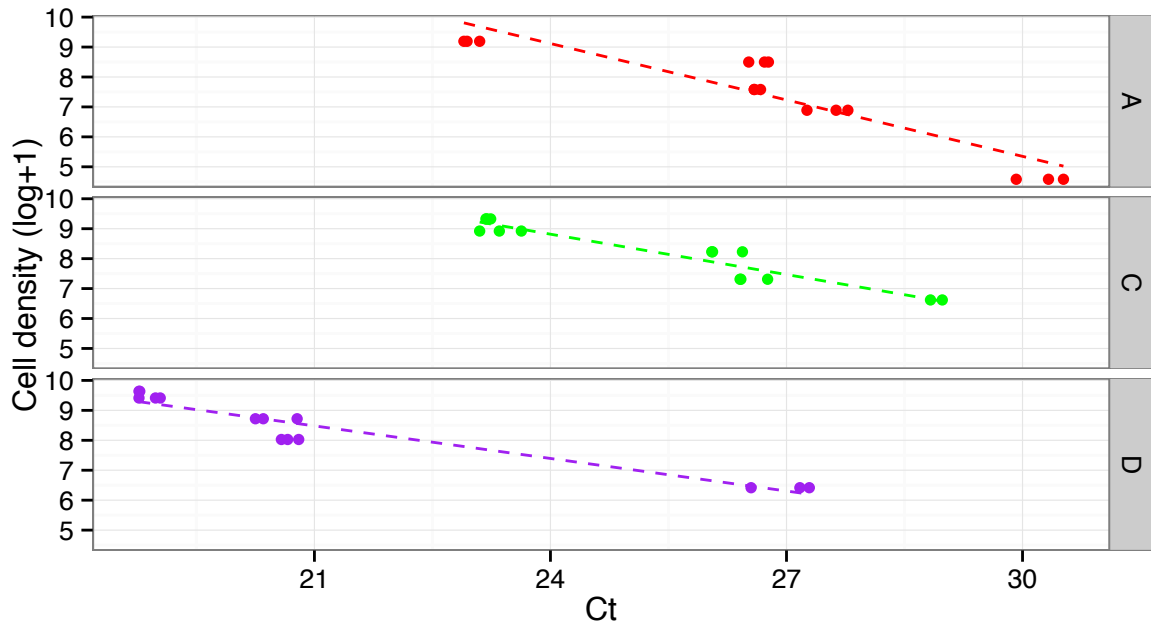

Supplement: Figure S2 — Standard curves for clade-specific primer sets for clades A, C and D corresponding to cell densities versus logarithmic 10 fold-dilution of isolated coral-symbiotic Symbiodinium. Each plot corresponds to individual Ct values obtained from three technical replicates. Primer efficiencies were deduced by linear regression: 1⋅1010 × e(−0.628x) (clade A, R2 = 0.85), 1⋅108 × e(−0.45x) (clade C, R2 = 0.91) and 4⋅106 × e(−0.363x) (clade D, R2 = 0.92). [file peerj-05-2856-s004.pdf]

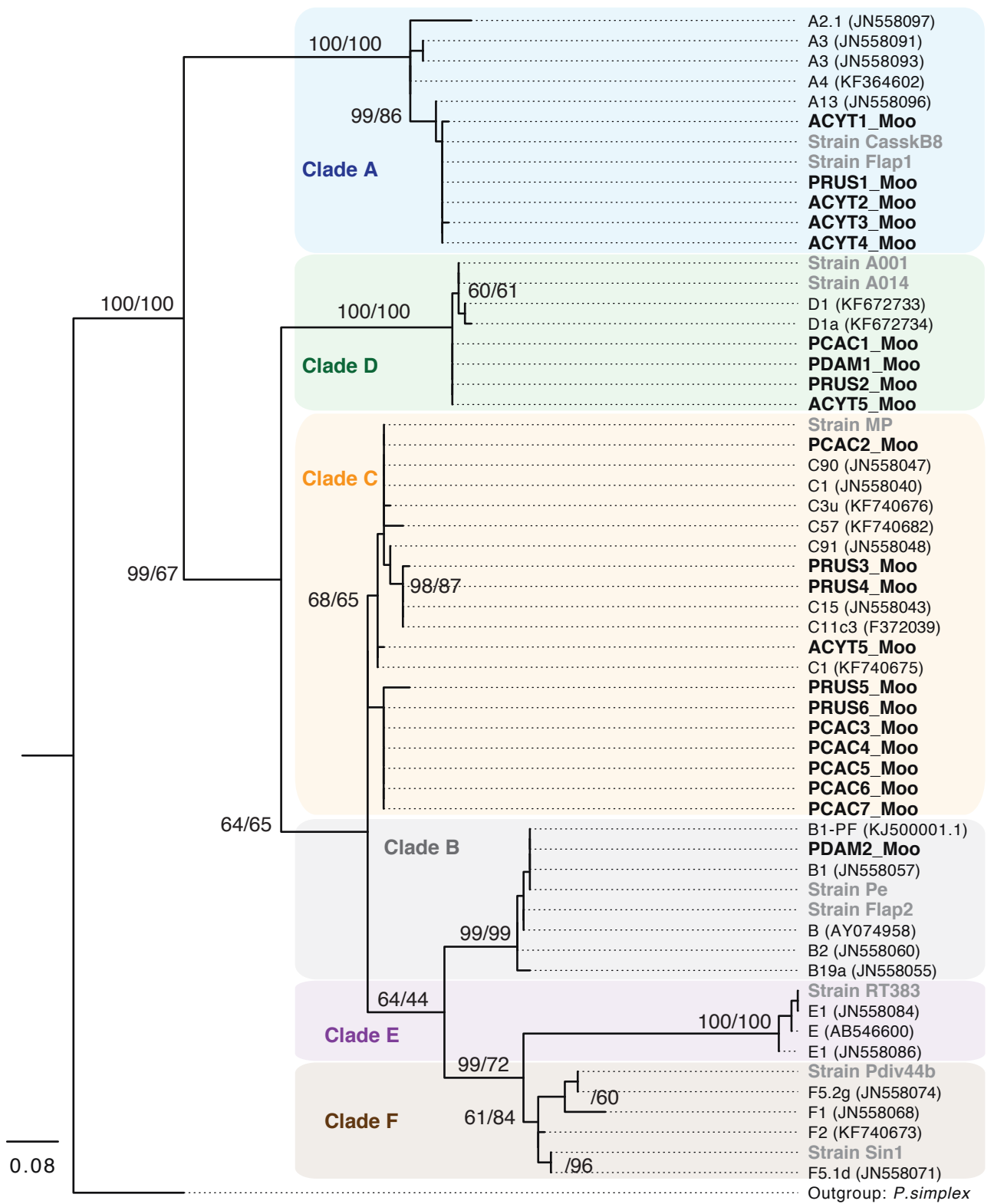

Supplement: Figure S3 — Phylogenetic tree of Symbiodinium clades A-F derived from bayesian analyses using sequences of 28S rDNA from Moorea (Moo; in black bold; sequences available in supplementary data: dataset 3), BURR collection strains described in Table S2 (in grey bold), and published genetic sequence from GenBank (regular font). Bayesian posterior probability (in percentage; first value) and Maximum Likelihood bootstrap support values (second value) are presented following the substitution model of Kimura 2-parameter with a proportion of invariable sites. [file peerj-05-2856-s005.pdf]

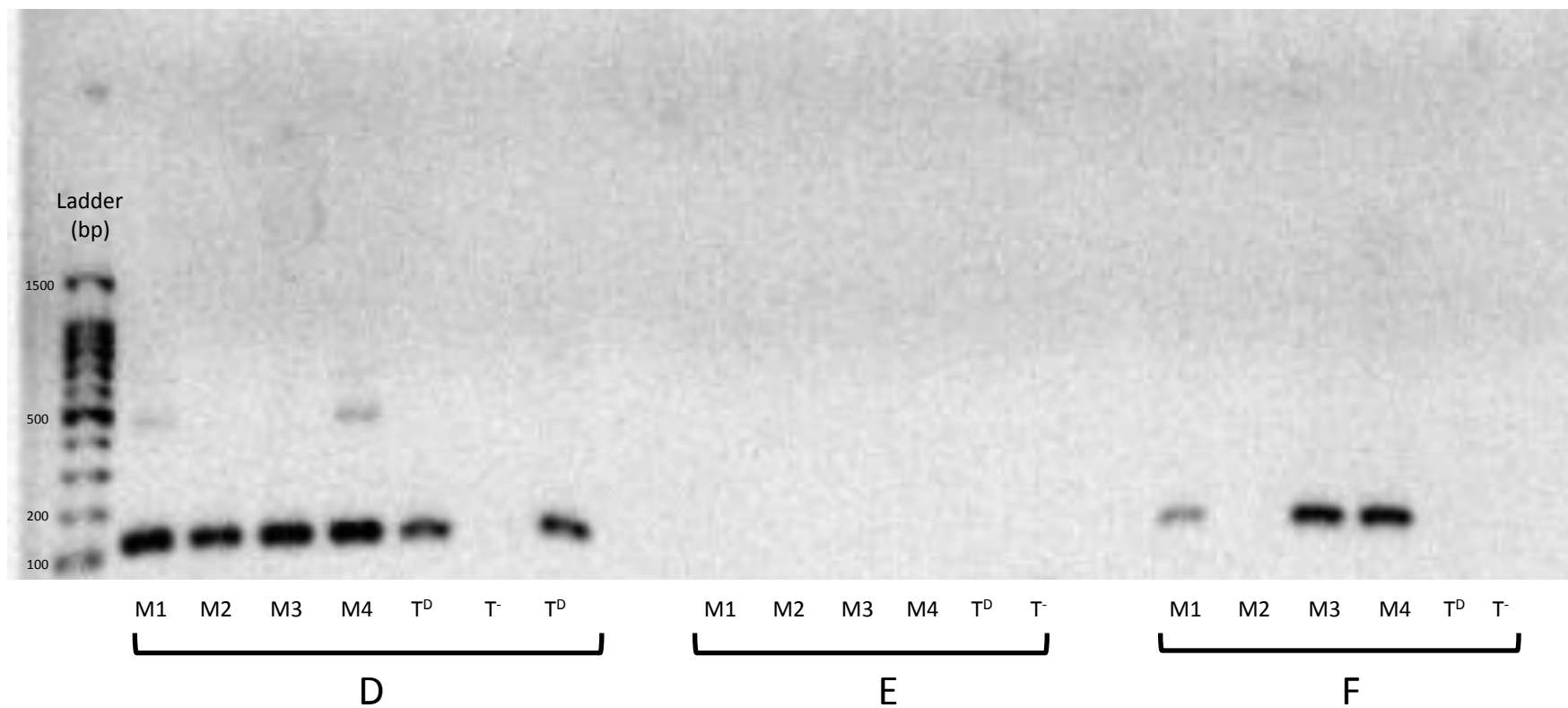

Supplement: Figure S4 — Presence of Symbiodinium clade C, E and F in seawater samples of Moorea. PCR amplifications of 28S rDNA using the primer sets clade-specific C, E and F (Yamashita et al., 2011) on DNA extracts from saturated 0.2 µm filters (filtration volume: 6-9 L) of 4 seawater samples (M1, M2, M3 and M4): T−: negative control (no DNA); TD: positive control (clade D DNA). A 100bp amplicon characterizes a positive amplification. Uppercase letters indicate the corresponding clade. [file peerj-05-2856-s006.pdf]
