# Supplementary material for: An updated assessment of Symbiodinium spp. that associate with common scleractinian corals from Moorea (French Polynesia) reveals high diversity among background symbionts and a novel finding of clade B
Source: PeerJ. 2017 Jan 5;5:e2856. doi: 10.7717/peerj.2856 (PMC5289445; doi:10.7717/peerj.2856)
Supplement: Table S1 [file peerj-05-2856-s007.docx]

**Title: Characteristic of primer sets used for qPCR**

**Legend:** Characteristics of primer sets used for identification of *Symbiodinium* clades associated to corals with qPCR assay on 28S amplicons (a) or coral DNA extract (b).

| **Primer** | | **Sequence (5'-3')** |  | **Melting temperature**  **(°C)** | **Genomic DNA** | | |  | **28S PCR products** | |
| --- | --- | --- | --- | --- | --- | --- | --- | --- | --- | --- |
|  |  |  | **Amplicon**  **(bp)** |  | **Specificity**^S2^ **(%)** | **Efficiency (%)** | **Sensitivity (Ct)** |  | **Efficiency (%)** | **Sensitivity (28S copy number)** |
|  | ***Symbiodinium*** |  |  |  |  |  |  |  |  |  |
| **A** | SymA-28S F^*^ | GATTGTGGCCTTTAGACATACTACC | 126 | 84.8 | 99.88 | 96.8 | 31.9 |  | 98.7 | 173 |
|  | SymA-28S R^*^ | CTCTGAGAGCAAGTACCGTGC |  |  |  |  |  |  |  |  |
| **B** | SymB-28S F^*^ | CACATGTCGTGCTGAGATTGC | 108 | 82.0 | 98.54 | 97.4 | 31.3 |  | 97 | 173 |
|  | SymB-28S R^*^ | CTCGCATGCTGAGAAACACTG |  |  |  |  |  |  |  |  |
| **C** | SymC-28S F^*^ | TTGCTGAGATTGCTGTAGGCT | 124 | 83.5 | 99.97 | 100 | 31.3 |  | 101.2 | 173 |
|  | SymC-28S R^*^ | TCCTCAAACAGGTGTGGC |  |  |  |  |  |  |  |  |
| **D** | SymD-28S F^*^ | AATGCTTGTGAGCCCTGGTC | 114 | 82.49 | 99.98 | 97.6 | 28.6 |  | 99.7 | 173 |
|  | SymD-28S R^*^ | AAGGCAATCCTCATGCGTATG |  |  |  |  |  |  |  |  |
| **E** | SymE-28S F^*^ | CGAGTTTTCACTAGCCTTGTGTG | 99 | 82.7 | 99.99 | 98.7 | 31.0 |  | 95.1 | 12 |
|  | SymE-28S R^*^ | AGCGTTGCAGCTGACGAG |  |  |  |  |  |  |  |  |
| **F** | SymF-28S F^*^ | ACAGATCTTGCTGAGATTGCTGTG | 143 | 85.5 | 99.21 | 95.7 | 31.6 |  | 100.7 | 173 |
|  | SymF-28S R^*^ | GAAGGCCGTCCTCAAACAGAC |  |  |  |  |  |  |  |  |
|  |  |  |  |  |  |  |  |  |  |  |
| ***Poyps*** | |  |  |  |  |  |  |  |  |  |
| UnivPolyp-18S F | | ATCGATGAAGAACGCAGCCA | 90 | 79.5 |  | 101.1 | 30.13 |  |  |  |
| UnivPolyp-18S R | | CAAGAGCGCCATTTGCGTTC |  |  |  |  |  |  |  |  |

* *Yamashita et al. 2011*

^S2^: Each primer set specificity has been estimated based on values of Ct indicated in the table S2
