# Supplementary material for: An updated assessment of Symbiodinium spp. that associate with common scleractinian corals from Moorea (French Polynesia) reveals high diversity among background symbionts and a novel finding of clade B
Source: PeerJ. 2017 Jan 5;5:e2856. doi: 10.7717/peerj.2856 (PMC5289445; doi:10.7717/peerj.2856)
Supplement: Table S2 — Values of Ct obtained by qPCR assays of the corresponding clade-specific primer sets on different DNAs issued from cultured Symbiodinium strains (clades A to F; BURR collection). Each DNA was tested at an equal amount of 10 ng per reaction. (-) means no amplification or unspecific amplifications based on dissociation curves analysis, and (*) symbiotic strains used in mix at same concentration and tested with each primer set. [file peerj-05-2856-s008.docx]

**Table S2**

| **ID strain** | **Host** | **Clade** | **Type**  (28S rDNA) | **Primer sets** | | | | | |
| --- | --- | --- | --- | --- | --- | --- | --- | --- | --- |
|  |  |  |  | **Sym A** | **Sym B** | **Sym C** | **Sym D** | **Sym E** | **Sym F** |
| CasskB8 * | Jellyfish | A | A13 | **19.36** | 33.17 | - | 30.18 | - | - |
| Flap1 | Anemone |  | A13 | **18.25** | na | na | na | na | na |
| Pe * | Coral | B | B1 | - | **18.29** | - | 31.37 | - | - |
| Flap2 | Anemone |  | B1 | na | **16.98** | na | na | na | na |
| Mp * | Jellyfish | C | C90 | - | 31.52 | **17.42** | 32.85 | - | - |
| A001 * | Coral | D | D1 | - | - | - | **15.79** | - | - |
| A014 | Coral |  | D1 | na | na | na | **17.19** | na | na |
| RT383 * | Anemone | E | E1 | - | - | 34.87 | - | **15.76** | - |
| Sin * | Soft coral | F | F5.1 | - | - | - | 29.03 | - | **18.76** |
| Pdiv44b | Coral |  | F5.2 | na | na | na | na | na | **18.36** |
| *Mix A-F (*)* | | |  | *28.12* | *27.84* | *27.05* | *24.88* | *24.83* | *27.35* |

na: no analysed
