# Supplementary material for: An updated assessment of Symbiodinium spp. that associate with common scleractinian corals from Moorea (French Polynesia) reveals high diversity among background symbionts and a novel finding of clade B
Source: PeerJ. 2017 Jan 5;5:e2856. doi: 10.7717/peerj.2856 (PMC5289445; doi:10.7717/peerj.2856)
Supplement: Data S2 — Symbiodinium 28S DNA sequences mentioned in the paper and used in the phylogenetic analysis (phylogenetic tree: Figure S3). [file peerj-05-2856-s009.docx]

***Symbiodinium* 28SrDNA sequences (~100 bp) obtained using the primer sets clade-specific of Yamashita et al. (2011)**

>PRUS1_Moo [*Symbiodinium* clade A]

CTA-TGCTCTGGTTGAGATTGCTGTAAATGGCTGCGACATCTGGGCTCTGGAACGCAAGTCCTTTGTCTGGCTT-AAGTAGTCGCTTGCAATGTGTCTCAGCTTGAACA

>ACYT2_Moo [*Symbiodinium* clade A]

CTA-TGCTCTGGTTGAGATTGCTGTAAATGGCTGCGACATCTGGGCTCTGGAACGCAAGTCCTTTGTCTGGCTT-AAGTAGTCGCTTGCAATGTGTCTCAGCTTGAACA

>ACYT1_Moo [*Symbiodinium* clade A]

CTA-TGCTCTGGTTGAGATTGCTGTAAATGGCTGCGACATCTGGGCTCTGGAACGCAAGTCCTTTGTCTGGCTT-AAGTAGTAGCTTGCAATGTGTCTCAGCTTGAACA

>ACYT3_Moo [*Symbiodinium* clade A]

CTC-TGCTCTGGTTGAGATTGCTGTAAATGGCTGCGACATCTGGGCTCTGGAACGCAAGTCCTTTGTCTGGCTT-AAGTAGTCGCTTGCAATGTGTCTCAGCTTGAACA

>ACYT4_Moo [*Symbiodinium* clade A]

CTA-TGCTCTGGTTGAGATTGCTGTAAATGGCTGCGACATCTGGGCTCTGGAACGCAAGTCCTTTGTCTGGCTT-AAGTAGTCGCTTGCAATGTGTCTCAGCTTGAACA

>PDAM2_Moo [*Symbiodinium* clade B]

TCACATGTCGTGCTGAGATTGCTGTGGGTCTTTGTGAGCCTTGAGCATGTAAGCGCAAGCTGACTGCTTATGTGTGAGCATTTACCCGCAGTGTTTCTCAGCATGCGAG

>PCAC2_Moo [*Symbiodinium* clade C]

CCA-ACGTCTTGCTGAGATTGCTGTAGGCTGCTGTGAGCCTTGGGCACATCAGCGCAAGCTGTGTGCTTAAGCG-GAATAGTTGTCTGCTGTGTTTCTTAGCTTGCGCG

>PRUS4_Moo [*Symbiodinium* clade C]

CCA-ACGTGTTGCTGAGATTGCTGTAGGCTGCTATGAGCCTTGGGCGCATCAGCGCAAGCTGTGTGCTTAAGCG-GAGTAGTTGTCTGCTGTGTTTCTTAGCTTGCGCG

>PRUS5_Moo [Symbiodinium clade C]

TCACATGTCGTGCTGAGATTGCTGTAGGCTGCTATGAGCCTTGGGCGCATCAGCTCAAGCTGTGTGCTTAAGCGTGAGTAGTTGTCTGCAGTGTTTCTCAGCATGCGAG

>PRUS3_Moo [*Symbiodinium* clade C]

CCA-ACGTGTTGCTGAGATTGCTGTAGGCTGCTATGAGCCTTGGGCGCATCAGCGCAAGCTGTGTGCTTAAGCG-GAGTAGTTGTGTGCTGTGTTTCTTAGCTTGCGCG

>ACYT5_Moo [*Symbiodinium* clade C]

CCA-ATGTGTTGCTGAGATTGCTGTAGGCTGCTGTGAGCCTTGGGCACATCAGCGCAAGCTGTGTGCTTCAGCG-GAATAGTTGTCTGCTGTGTTTCTTAGCTTGCGTG

>PCAC1_Moo [*Symbiodinium* clade D]

CCA-ATGTCTTGCTGAGATTGCTGCCAATGCTTGTGAGCCCTGGTCATTAAAGCGCAAGCTTCTTGTCTAGGAG-GAGTTGGCATTTGTAGTGCTTCTTAGCTTGCGCG

>PDAM1_Moo [*Symbiodinium* clade D]

CCA-ATGTCTTGCTGAGATTGCTGCCAATGCTTGTGAGCCCTGGTCATTAAAGCGCAAGCTTCTTGTCTAGGAG-GAGTTGGCATTTGTAGTGCTTCTTAGCTTGCGCG

>PRUS2_Moo [*Symbiodinium* clade D]

CCA-ATGTCTTGCTGAGATTGCTGCCAATGCTTGTGAGCCCTGGTCATTAAAGCGCAAGCTTCTTGTCTAGGAG-GAGTTGGCATTTGTAGTGCTTCTTAGCTTGCGCG

>ACYT5_Moo [*Symbiodinium* clade C]

CCA-ATGTCTTGCTGAGATTGCTGCCAATGCTTGTGAGCCCTGGTCATTAAAGCGCAAGCTTCTTGTCTAGGAG-GAGTTGGCATTTGTAGTGCTTCTTAGCTTGCGCG

>PRUS6_Moo [*Symbiodinium* clade C]

TCACATGTCGTGCTGAGATTGCTGTAGGCTGCTGTGAGCCTTGGGCACATCAGCGCAAGCTGTGTGCTTAAGCGTGAATAGTTGTCTGCAGTGTTTCTCAGCATGCGAG

>PCAC3_Moo [*Symbiodinium* clade C]

TCACATGTCGTGCTGAGATTGCTGTAGGCTGCTGTGAGCCTTGGGCACATCAGCGCAAGCTGTGTGCTTAAGCGTGAATAGTTGTCTGCAGTGTTTCTCAGCATGCGAG

>PCAC4_Moo [*Symbiodinium* clade C]

TCACATGTCGTGCTGAGATTGCTGTAGGCTGCTGTGAGCCTTGGGCACATCAGCGCAAGCTGTGTGCTTAAGCGTGAATAGTTGTCTGCAGTGTTTCTCAGCATGCGAG

>PCAC5_Moo [*Symbiodinium* clade C]

TCACATGTCGTGCTGAGATTGCTGTAGGCTGCTGTGAGCCTTGGGCACATCAGCGCAAGCTGTGTGCTTAAGCGTGAATAGTTGTCTGCAGTGTTTCTCAGCATGCGAG

>PCAC6_Moo [*Symbiodinium* clade C]

TCACATGTCGTGCTGAGATTGCTGTAGGCTGCTGTGAGCCTTGGGCACATCAGCGCAAGCTGTGTGCTTAAGCGTGAATAGTTGTCTGCAGTGTTTCTCAGCATGCGAG

>PCAC7_Moo [*Symbiodinium* clade C]

TCACATGTCGTGCTGAGATTGCTGTAGGCTGCTGTGAGCCTTGGGCACATCAGCGCAAGCTGTGTGCTTAAGCGTGAATAGTTGTCTGCAGTGTTTCTCAGCATGCGAG
